# From Hype to Implementation: Embedding GPT-4o in Medical Education

**DOI:** 10.2196/79309

**Published:** 2025-10-15

**Authors:** Sumaia Sabouni, Mohammad-Adel Moufti, Mohamed Hassan Taha

**Affiliations:** 1 Department of Data Science and Artificial Intelligence, Faculty of Information Technology, Al-Ahliyya Amman University, Amman, Jordan; 2 College of Dental Medicine, University of Sharjah, Sharjah, 27272, United Arab Emirates, 971 65057314; 3 Medical Education Center, University of Sharjah, Sharjah, United Arab Emirates; 4 College of Medicine, University of Sharjah, Sharjah, United Arab Emirates

**Keywords:** multimodal generative pretraining transformers, GPT-4o, artificial intelligence, interactive learning, medical education

## Abstract

The release of GPT-4 Omni (GPT-4o), an advanced multimodal generative artificial intelligence (AI) model, generated substantial enthusiasm in the field of higher education. However, one year later, medical education continues to face significant challenges, demonstrating the need to move from initial experimentation with the integration of multimodal AIs in medical education toward meaningful integration. In this Viewpoint, we argue that GPT-4o’s true value lies not in novelty, but in its potential to enhance training in communication skills, clinical reasoning, and procedural skills by offering real-time simulations and adaptive learning experiences using text, audio, and visual inputs in a safe, immersive, and cost-effective environment. We explore how this innovation has made it possible to address key medical educational challenges by simulating realistic patient interactions, offering personalized feedback, and reducing educator workloads and costs, where traditional teaching methods struggle to replicate the complexity and dynamism of real-world clinical scenarios. However, we also address the critical challenges of this approach, including data accuracy, bias, and ethical decision-making. Rather than seeing GPT-4o as a replacement, we propose its use as a strategic supplement, scaffolded into curriculum frameworks and evaluated through ongoing research. As the focus shifts from AI novelty to sustainable implementation, we call on educators, policymakers, and curriculum designers to establish governance mechanisms, pilot evaluation strategies, and develop faculty training. The future of AI in medical education depends not on the next breakthrough, but on how we integrate today’s tools with intention and rigor.

## Introduction

The impact of artificial intelligence (AI) within health care continues to expand, ranging from diagnostic imaging to medical training [1]. Its integration into medical education offers promising solutions to numerous challenges, particularly the need for substantial resources to effectively train students in competencies like communication, clinical reasoning, and procedural skills. AI tools offer scalable, low-resource alternatives that can help bridge these gaps.

In 2024, OpenAI released GPT-4 Omni (GPT-4o), a multimodal model combining text, image, and audio processing. Unlike text-only systems, GPT-4o supports more dynamic interactions, such as engaging in real-time, human-like conversations, interpreting visual content, and performing realistic voice-based simulations. GPT-4o received widespread attention within medical education due to its multimodal capabilities enabling realistic simulations that extend to procedural skill assessment and interactive case analyses. Early demonstrations highlight GPT-4o’s ability to simulate patient interactions [2], visually identify medications [3], and undertake medical examinations with higher accuracy than GPT-4 and GPT-3.5 [4,5].

However, one year after its release, there is clearly a slow pace of adoption and implementation remains limited [6]. Although some institutions have experimented with GPT-4o integration [7] and others analyzed medical students’ attitudes toward this integration [8], sustained adoption within formal curricula has lagged.

This Viewpoint highlights how GPT-4o shows promise as a relevant tool to enhance medical education and address many of its challenges, despite the initial hype fading. We examine its strengths in simulating patient interactions, increasing equity due to low-resource deployment, and reducing educator workload. We also address concerns including accuracy, bias, ethical reasoning, and digital access. Our aim is not to present GPT-4o as a cure-all, but to advocate for a structured, research-informed pathway for integrating such tools meaningfully into medical training. Although this discussion focuses on general medical education, specific considerations for dental medicine and other health disciplines warrant future exploration.

## Educational Challenges Addressed by GPT-4o

Here are some of the educational challenges addressed by GPT-4o:

1. Communication skills: Developing interpersonal communication is vital for effective clinical practice. Traditional role-playing methods, reliant on patients or actors, are resource-intensive and challenging to standardize and scale. GPT-4o–generated virtual patients simulate diverse and adaptive interactions, fostering essential skills such as delivering bad news and providing culturally sensitive care. By creating realistic, immersive environments, GPT-4o aligns with situated learning theory, enabling learners to acquire practical skills in context.
2. Clinical reasoning: Diagnostic and intervention decision-making requires iterative exposure to varied scenarios. Static case-based learning often fails to engage learners dynamically. GPT-4o generates interactive, context-rich cases tailored to learner actions, promoting experiential learning [9] and hypotheticodeductive reasoning [10]. Feedback is immediate and adaptive, allowing users to refine diagnostic strategies in real time. These features advance cognitive load management, enhancing learning efficiency.
3. Essay-based assessments: Essay writing fosters critical thinking and provides a platform for expressing individual ideas. Despite these benefits, educators often avoid using essay writing due to the time required for grading. GPT-4o automates the assessment of essay responses, providing detailed feedback on reasoning, structure, and language [11]. This automation reduces educator workloads, encourages higher-order assessments, and supports personalized student development. However, overreliance on automated grading may overlook nuanced clinical reasoning or professional reflection, underscoring the need for educator oversight [12] Additionally, some research suggests that students may perceive AI-generated feedback as impersonal, leading to reduced engagement or trust [11].
4. Procedural skills: Skill acquisition in procedures relies on hands-on practice, detailed feedback, and in-person, one-on-one observation and assessment. GPT-4o’s ability to produce realistic simulations and analyze video inputs offers scalable solutions for performance evaluation. Future integration with augmented reality and virtual reality technologies could facilitate immersive training experiences, replicating clinical environments for enhanced procedural competence. However, the concept of fidelity, including how realistic and believable simulations appear to learners, is critical. Low-fidelity simulations risk invoking the “uncanny valley” effect, where interactions feel unnatural and may reduce engagement [13].

## Potential Benefits

### For Learners

GPT-4o supports ongoing skill development by offering, personalized instant performance evaluation and feedback. Due to its realistic clinical simulations, students are exposed to a wide range of medical cases and can practice clinical reasoning in a realistic and practical way. Learners can practice breaking bad news to virtual patients, interpret multimodal data such as radiological images or heart sounds, and receive adaptive feedback tailored to their level. As a result, students can develop their confidence in a secure, flexible, and nonjudgmental learning environment.

### For Educators

GPT-4o helps ease the workload by performing time-consuming tasks such as writing patient scenarios, setting assessments, marking essays, and providing personalized feedback on student performance. This gives educators more time to focus on tasks such as student support and course development. Additionally, seeing as GPT-4o is already pretrained, there is no need for heavy investment in custom AI tools, making it easier and quicker for institutions to adopt. Nevertheless, institutions should address faculty resistance by providing hands-on workshops, co-designing AI use cases with educators, and sharing evidence from pilot studies to build confidence in its implementation.

### Global Accessibility

Due to GPT-4o being cloud-based, it does not require costly infrastructure, making it accessible to both learners and educators in low-resource settings. This helps democratize medical education and training and results in a more even distribution of health care expertise globally [14].

## Challenges and Considerations

Although AI holds promise in medical education, there are also some challenges and considerations:

1. Accuracy: AI programs like GPT-4o may generate errors or “hallucinations” [15]. To overcome this, the output of such tools requires continuous supervision and validation to ensure their reliability.
2. Data privacy: To uphold confidentiality and ethical standards, learner performance data must be protected. This may be achieved through the adoption of strict data security measures by educational establishments. However, implementing strict data security is complex, requiring closed systems, robust encryption, and governance frameworks. There are trade-offs between using real learner data, which raises confidentiality concerns, and synthetic data, which may lack realism.
3. Bias: Training data biases associated with gender, ethnicity, or socioeconomic status may influence AI outputs. As a result, initiatives to reduce these biases are essential, especially since AI tools are being used more and more to deliver and filter health care information, where transparency and trust are essential [16]. Emerging frameworks like Fairlearn and AI Fairness 360 offer tools to audit and mitigate bias in AI systems, supporting fairer educational outcomes [17].
4. Ethics and professionalism: Although GPT-4o performs very well in simulations, professionalism or complex ethical reasoning may be more challenging for it [12]. These limitations highlight the importance of human oversight and complementary teaching methods.
5. Access: Despite its general affordability, access to GPT-4o still depends on a stable internet connection, potentially limiting its reach in underserved regions with poor connectivity [18]. Addressing this challenge requires exploring offline or low-bandwidth alternatives to ensure a broader reach.

## Next Steps

Emerging empirical studies are beginning to validate GPT-4o’s role in medical education. For example, a pilot by Öncü et al [2] used GPT-4o as a virtual standardized patient to simulate complex communication and crisis scenarios, demonstrating high learner engagement and feasibility in training clinical reasoning. Bicknell et al [19] evaluated GPT-4o across 4 national licensing exams, where it achieved a 90.4% accuracy rate, surpassing GPT-4 and average medical student performance. Similarly, Zhong et al [20] benchmarked GPT-4o for rare disease diagnosis using multilingual clinical data, with the model achieving the highest diagnostic accuracy among tested large language models. These studies reinforce the potential of GPT-4o to enhance assessment, simulation, and clinical education, while also underscoring the need for rigorous longitudinal evaluation frameworks.

Future research should assess how effectively GPT-4o enhances learner competencies like critical thinking, decision-making, and procedural skills. Evaluation frameworks should include multimodal fidelity measures, learner satisfaction, performance metrics, and longitudinal tracking of outcomes such as clinical preparedness. These metrics will help assess the educational impact of GPT-4o in realistic, complex learning environments. Additionally, GPT-4o could support interprofessional education by simulating collaborative scenarios between medical, nursing, and dental students. Such integration may enhance communication, teamwork, and understanding across health disciplines, particularly in complex patient care settings.

To further expand its uses, GPT’s multimodal capabilities could be extended to interpret video and audio data, such as echo scans and heart sounds. To guarantee that implementation is ethical, scalable, and in line with curriculum objectives, cooperation between AI developers, educators, and legislators is equally vital. Addressing challenges such as accuracy, bias, and accessibility challenges will ensure equitable benefits for all learners. Even though GPT-4o is a significant step forward, its use in medical education should be guided by research and specific learning aims. To offer useful assistance for adoption, we propose a set of initial steps for institutions to implement GPT-4o (Textbox 1).

Textbox 1. **Recommendations for institutions considering GPT-4o integration.**

1. Begin with a small-scale pilot project focusing on a single competency (eg, communication).
2. Provide prompt engineering training for faculty members.
3. Develop ethical guidelines for student use.
4. Incorporate artificial intelligence literacy into medical curricula.
5. Collaborate with technology experts to conduct longitudinal assessments of performance.
6. Design simulated scenarios that support interprofessional education (eg, medical, dental, and nursing collaboration).
7. Address faculty concerns through workshops, co-designed implementation strategies, and sharing evidence from early adopters.

## Conclusion

The integration of GPT-4o into medical education represents a promising shift. It not only tackles long-standing challenges by offering scalable, immersive training solutions, but its multimodal nature also results in it offering advantages over prior models in certain contexts. Consequently, both educators and learners globally stand to benefit as access to quality education becomes more equitable. However, for this integration to be effective, medical institutions must move past experimentation through pilot studies and toward embedding into formal curricula. Finally, GPT-4o must not be viewed as a quick fix but rather as a tool that demands thoughtful design, evaluation, and governance, and its integration should be guided by rigorous research, ethical considerations, and faculty collaboration.

## Abbreviations

AI: artificial intelligence
GPT-4o: GPT-4 Omni
